# Serum Endothelin-1 in Suspected Acute Pulmonary Embolism: A Prospective Study

**DOI:** 10.3390/jcdd13090448

**Published:** 2026-09-09

**Authors:** Mahmut Murat, Mustafa Safa Pepele, Serdar Derya, Gökay Sülü, Neslihan Yücel, Cuma Mertoğlu, Hasan Cengizhan Katırcı, Ahmet Burak Avcu

**Affiliations:** 1 Department of Emergency Medicine, Mersin City Training and Research Hospital, 33240 Mersin, Türkiye; mahmutmurat5@gmail.com; 2 Department of Emergency Medicine, Inonu University School of Medicine, 44280 Malatya, Türkiye; safa.pepele@inonu.edu.tr (M.S.P.); serdar.derya@inonu.edu.tr (S.D.); neslihan.yucel@inonu.edu.tr (N.Y.); 3 Department of Emergency Medicine, Yesilyurt Hasan Calik District State Hospital, 44915 Malatya, Türkiye; gkysl01@gmail.com; 4 Department of Biochemistry, Inonu University School of Medicine, 44280 Malatya, Türkiye; cuma.mertoglu@inonu.edu.tr (C.M.); hasan.katirci@inonu.edu.tr (H.C.K.); 5 Department of Public Health, Inonu University School of Medicine, 44280 Malatya, Türkiye

**Keywords:** biomarkers, d-dimer, pulmonary embolism

## Abstract

Background: We evaluated the diagnostic discrimination of serum endothelin-1 (ET-1), copeptin, and chemerin in adults evaluated for acute pulmonary embolism (PE). Methods: This prospective, single-center diagnostic study included 65 adults with CTPA-confirmed PE and 64 CTPA-negative controls recruited from the same emergency-department source population of patients evaluated for suspected PE. Controls were consecutively enrolled among dyspneic patients in whom CTPA excluded PE; no individual matching was used. Biomarkers were compared with D-dimer and the Wells score. Results: ET-1 was higher in the PE group (median, 12.96 vs. 6.41 pg/mL; *p* < 0.001) and showed moderate discrimination (AUROC, 0.731; 95% CI, 0.644–0.818), but was inferior to D-dimer (AUROC, 0.905; 95% CI, 0.852–0.958). Adding ET-1 to D-dimer changed the AUROC from 0.905 to 0.923 (DeLong *p* = 0.128), and adding ET-1 to Wells plus D-dimer changed it from 0.966 to 0.971 (DeLong *p* = 0.224). An age-adjusted D-dimer threshold preserved sensitivity (96.9%) and increased specificity from 48.4% to 56.3% relative to 0.50 mg/L FEU. Conclusions: ET-1 was associated with acute PE in this selected sample but did not materially improve discrimination beyond established comparators. Because the near-ceiling Wells–D-dimer model and selected sampling frame limit inference, external validation in an unselected diagnostic cohort is required.

## 1. Introduction

Acute pulmonary embolism (PE) is a common and potentially fatal cardiovascular emergency whose diagnosis remains challenging because its symptoms and signs overlap with those of many cardiopulmonary disorders [1,2]. Contemporary pathways combine clinical pre-test probability with D-dimer testing and confirmatory imaging, most commonly computed tomographic pulmonary angiography (CTPA) [1]. Although D-dimer is highly sensitive, its limited specificity can lead to substantial imaging use [3]. A clinically useful new biomarker would therefore need to add information beyond the established clinical probability–D-dimer pathway, rather than merely separating selected cases from controls [4].

This unmet need has sustained interest in circulating biomarkers that reflect the endothelial, neurohormonal, and inflammatory consequences of acute pulmonary vascular obstruction, thereby providing information complementary to clinical scores and imaging [5,6]. Beyond mechanical obstruction, acute PE involves thrombo-inflammatory processes in which endothelial activation, platelet–leukocyte crosstalk, and cytokine signaling may amplify the hemodynamic insult [7,8]. Within this framework, we evaluated three mechanistically distinct circulating candidates (endothelin-1, copeptin, and chemerin) with CTPA as the prespecified reference standard.

Endothelin-1 (ET-1) is a potent endogenous vasoconstrictor produced predominantly by the vascular endothelium; the lung is a major site of both ET-1 synthesis and clearance [9,10]. ET-1 is implicated in pulmonary vascular pathobiology, including pulmonary hypertension and acute lung injury [11,12]. Experimental pulmonary thromboembolism models demonstrate local ET-1 upregulation and improved hemodynamics after endothelin receptor blockade [13].

Clinical evidence across thromboembolic pulmonary disease is heterogeneous. Sofia et al. reported increased circulating ET-1 during acute PE [14]. In chronic thromboembolic pulmonary hypertension, Benchenouf et al. demonstrated heightened circulating and pulmonary microvascular ET-1-pathway activity [15], while Totaro et al. observed reductions in circulating ET-1 after pulmonary endarterectomy [16]. These chronic-disease findings support biological plausibility but cannot establish diagnostic performance at acute presentation. Conversely, Kostrubiec et al. found no elevation of ET-1 in acute PE [17]. Thus, the association of ET-1 with acute PE at emergency-department presentation and its diagnostic discrimination remain uncertain.

Copeptin has been linked to right-ventricular dilatation, severity, and prognosis in acute PE [18,19,20], but its diagnostic performance depends strongly on the comparison group. Studies using healthy controls have reported moderate-to-good discrimination [21,22], whereas studies using symptomatic emergency-department controls have found little or no diagnostic value [23,24]. Copeptin, the stable C-terminal fragment of the arginine–vasopressin precursor, is a surrogate marker of generalized acute neurohormonal stress [25,26]. This non-specific response may explain the inconsistency across comparison groups.

Chemerin is a chemoattractant adipokine linking metabolic dysregulation to endothelial inflammation, leukocyte recruitment, and vascular dysfunction [27,28]. It activates endothelial NF-κB signaling and promotes monocyte–endothelial adhesion [29], mechanisms plausibly relevant to thrombo-inflammatory responses. Clinical evidence for chemerin as a diagnostic biomarker in acute PE remains sparse [4].

We therefore evaluated serum ET-1, copeptin, and chemerin in a prospectively enrolled sample of adults with CTPA-confirmed PE and symptomatic CTPA-negative controls drawn from the same emergency-department source population. The primary objective was to estimate the diagnostic discrimination of the three candidate biomarkers. Secondary exploratory objectives were to compare them with D-dimer and the Wells score, assess ET-1 added to D-dimer alone and to Wells plus D-dimer, and examine associations with right-ventricular dysfunction, PESI-based severity, and in-hospital mortality.

## 2. Materials and Methods

### 2.1. Study Design, Setting, and Participants

This was a prospective, single-center diagnostic study in which patients evaluated for suspected PE and CTPA-negative controls were recruited from the same emergency-department source population. This study was conducted at Inonu University Turgut Ozal Medical Center, a tertiary university hospital in Malatya, Türkiye, between June 2024 and February 2025. Reporting was guided by the STARD 2015 statement [30].

Adults aged ≥18 years undergoing emergency-department evaluation for suspected acute PE were eligible. Participants with CTPA-confirmed acute PE comprised the PE group. The controls were consecutive patients presenting with dyspnea who were evaluated for suspected PE and in whom technically adequate CTPA excluded PE. Controls were not individually matched to PE cases, and no fixed-pair, propensity-score, age- or sex-based quota, or other formal matching method was used. At presentation, an additional study blood tube was collected from every enrolled participant before initiation of treatment and before the final CTPA result was known; CTPA subsequently defined the groups used for analysis. The enrolled sample was assembled for between-group comparison and was not intended to estimate PE prevalence or diagnostic performance in an unselected emergency-department population.

Patients were excluded if they had conditions likely to substantially alter their copeptin, endothelin-1, or chemerin concentrations, including hyponatremia or diabetes insipidus, severe decompensated heart failure, acute coronary syndrome, hemorrhagic or septic shock, acute cerebrovascular disease, renal failure, acute exacerbation of chronic obstructive pulmonary disease, acute lower respiratory tract infection, or paraneoplastic syndromes, including syndrome of inappropriate antidiuretic hormone secretion.

**Figure 1:**
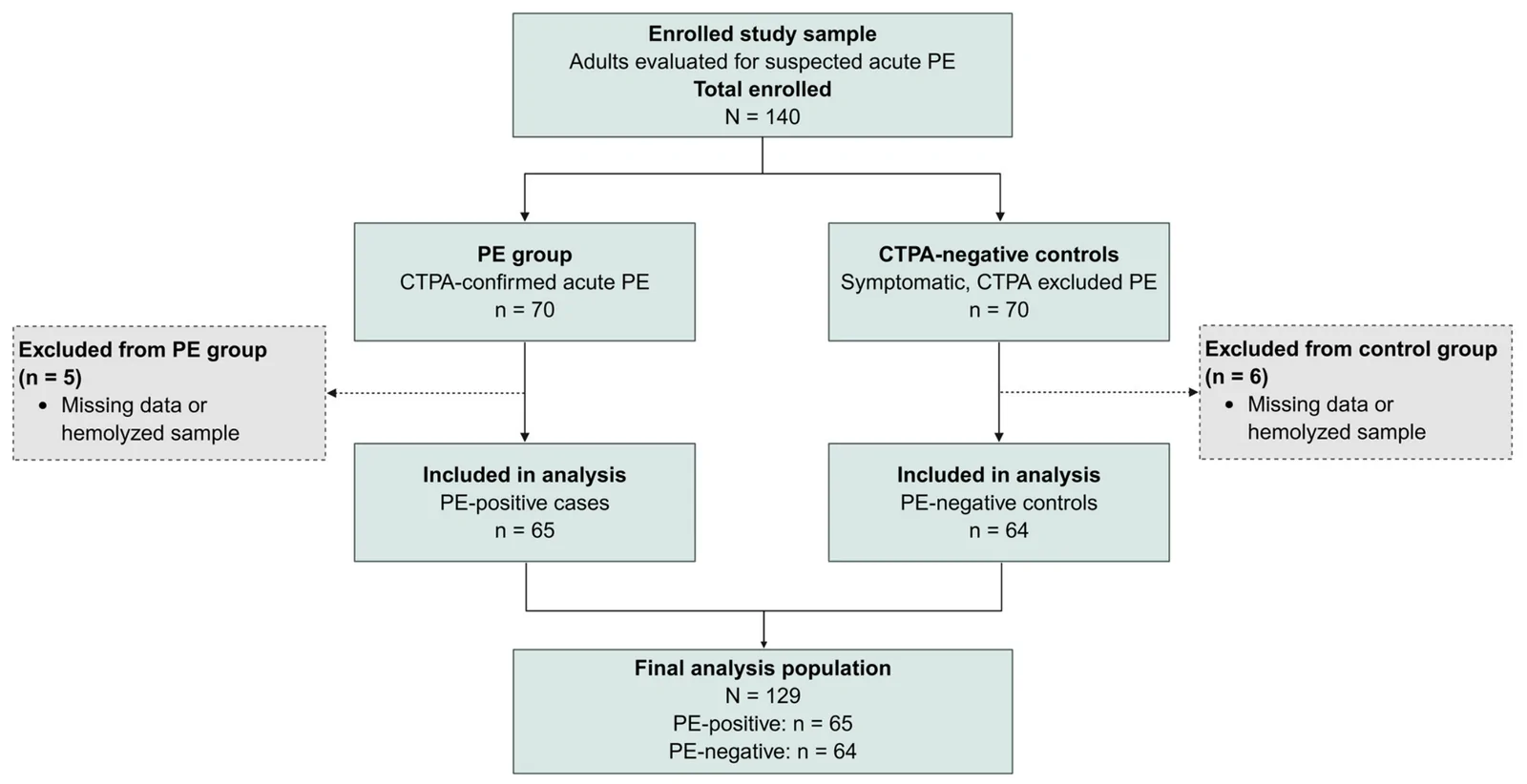
Flow of the enrolled study sample. Note. The diagram begins with the enrolled study sample rather than all patients screened in the emergency department, and therefore depicts within-study flow, not population screening flow. The enrolled sample comprised 70 participants with CTPA-confirmed PE and 70 consecutive dyspneic controls in whom CTPA excluded PE. The precise stopping rule that produced the initially balanced groups was not available for verification. Five participants in the PE group and six controls were excluded because of missing data or hemolyzed samples. All 129 analyzed participants underwent CTPA; acute PE was confirmed in 65 and excluded in 64 controls. The observed group sizes should not be interpreted as PE prevalence or as evidence of age- or sex-based matching.

Participants were enrolled prospectively, and the final study-group assignment was made after CTPA. Figure 1 summarizes the flow of the enrolled study sample, subsequent exclusions, and final analysis set. The *N* = 140 denominator represents the enrolled study sample rather than all patients screened in the emergency department. The precise stopping rule that produced the initially balanced groups was not available for verification and is not inferred. This study was conducted in accordance with the Declaration of Helsinki and was approved by the Health Sciences Scientific Research Ethics Committee of Inonu University (5 June 2024; approval no. 2024/67). Written informed consent was obtained from all participants before enrollment.

### 2.2. Reference Standard

All 129 analyzed participants underwent computed tomographic pulmonary angiography (CTPA), which served as the sole reference standard. CTPA was performed using a CT scanner (Siemens Healthineers, Forchheim, Germany). Acute PE was defined as an intraluminal filling defect in a main, lobar, segmental, or subsegmental pulmonary artery on technically adequate CTPA. Acute PE was confirmed by CTPA in 65 participants and excluded by technically adequate CTPA in 64 symptomatic controls. No participant in the analysis was classified on the basis of ventilation/perfusion scintigraphy.

CTPA examinations were interpreted by radiologists who were unaware of the subsequently measured research-biomarker results. The radiologists had access to routine clinical information as part of usual care. Participants without a technically adequate definitive CTPA result were not included in the diagnostic accuracy analysis.

### 2.3. Biomarker Measurements

The index tests targeted serum copeptin, endothelin-1 (ET-1), and chemerin concentrations. At presentation, an additional peripheral venous blood tube was collected from every enrolled participant for study-biomarker measurements before initiation of treatment and before the final CTPA result was known. Sampling time of day, body position, preceding physical activity, and duration of rest were not standardized or prospectively recorded, reflecting pragmatic emergency-care conditions. Samples were processed for batch analysis according to the study laboratory protocol; however, detailed records of storage conditions and duration, freeze–thaw cycles, and lot- or plate-level quality-control results were not available for the present analysis.

Biomarkers were measured in duplicate using commercially available ELISA kits from Reed Biotech Ltd. (Wuhan, China), according to the manufacturer’s instructions. The kit codes were RE1470H for copeptin, RE1036H for ET-1, and RE1387H for chemerin. The manufacturer-reported limits of detection were 18.75 pg/mL for copeptin, 0.75 pg/mL for ET-1, and 0.10 ng/mL for chemerin; their corresponding measurement ranges were 31.25–2000 pg/mL, 1.25–80 pg/mL, and 0.16–10 ng/mL, respectively. The kit documentation available to the investigators reported intra-assay coefficients of variation < 10%; lot-specific inter-assay precision and complete plate-level quality-control records for the study runs were not available for retrospective verification.

Laboratory personnel were blinded to the clinical data, imaging results, and final diagnostic classification. Patient and control samples were distributed across assay plates to reduce batch effects. Biomarker data were merged with clinical and imaging data only after completion of laboratory analyses.

### 2.4. Clinical Data and Comparator Measures

Demographic data, comorbidities, venous thromboembolism risk factors, presenting symptoms, vital signs, routine laboratory results, and imaging findings were recorded prospectively and cross-checked against the electronic hospital information system. The Wells score was calculated prospectively by the evaluating physician during the initial assessment, entered contemporaneously, and completed before CTPA interpretation and final diagnostic classification; the subjective item reflected the physician’s pre-imaging judgment. Modified Geneva, PESI, sPESI, and PESI risk classes were available only for PE-confirmed participants and were used only in descriptive or exploratory within-PE analyses, not as diagnostic comparators.

Routine D-dimer results were obtained from clinical testing using the INNOVANCE D-Dimer assay (Siemens Healthcare Diagnostics Products GmbH, Marburg, Germany) on a CN-6000 analyzer (Sysmex Corporation, Kobe, Japan) and were reported in mg/L fibrinogen-equivalent units (FEU). The manufacturer’s routine decision threshold was 0.50 mg/L FEU. The exact timing of routine D-dimer sampling relative to treatment initiation was not separately verified; therefore, no treatment-timing assumption was applied to D-dimer.

Transthoracic echocardiography was performed in all 129 participants during the index clinical evaluation. The operational definition of RV dysfunction was an end-diastolic right-ventricular-to-left-ventricular diameter ratio (RV/LV) ≥ 1.0 or tricuspid annular plane systolic excursion (TAPSE) < 16 mm; either criterion was sufficient when documented [1]. RV free-wall hypokinesia, interventricular septal flattening, and the McConnell sign were considered supportive findings and were not sufficient alone. The analytical dataset retained the resulting binary RV-dysfunction status but not the individual numeric RV/LV or TAPSE measurements or source images for central re-reading; consequently, the component thresholds could not be re-applied or independently checked at the participant level. Echocardiographers were unaware of subsequently measured research-biomarker results but had access to routine clinical information. Previous echocardiograms were not systematically available; therefore, the binary index-TTE endpoint could not always distinguish acute PE-related strain from pre-existing RV dysfunction.

The study data-collection instrument did not prospectively capture height or weight; BMI or obesity status; final discharge diagnoses among controls; complete variables required for ESC acute-PE risk classification; obstructive-shock status; or use of systemic thrombolysis or other advanced reperfusion therapies, including catheter-directed treatment and surgical embolectomy. These variables were unavailable for analysis and were neither imputed nor retrospectively inferred from incomplete records.

### 2.5. Handling of Missing and Indeterminate Results

All three index-biomarker values were present for the 129 analyzed participants. The analytic dataset contained numerical concentrations both within and outside manufacturer-reported measurement ranges. Primary analyses used the reported concentrations. Robustness was assessed by repeating group comparisons and AUROC analyses after censoring values at the reported lower and upper measurement boundaries and after excluding out-of-range observations.

### 2.6. Statistical Analysis

Statistical analyses were performed in R (version 4.6.0; R Foundation for Statistical Computing, Vienna, Austria). Continuous variables were summarized as mean ± standard deviation or median with interquartile range, according to the distributional characteristics assessed using the Shapiro–Wilk test and visual inspection of histograms and Q-Q plots. Categorical variables were summarized as counts and percentages. Between-group comparisons used the independent-sample *t* test or Mann–Whitney U test for continuous variables and Pearson’s χ^2^ test without continuity correction or Fisher’s exact test for categorical variables, as appropriate.

The primary diagnostic endpoint was the area under the receiver operating characteristic curve (AUROC) for each continuous biomarker in discriminating CTPA-confirmed PE from the symptomatic CTPA-negative controls. AUROCs and 95% confidence intervals were estimated using the DeLong method, and correlated AUROCs were compared using the DeLong test. Because no validated diagnostic thresholds exist for the candidate biomarkers in acute PE, candidate-biomarker concentrations were analyzed primarily as continuous variables; exploratory cutoffs were selected by maximizing Youden’s J statistic in the study sample. For D-dimer, operating characteristics were also estimated at the routine fixed threshold of 0.50 mg/L FEU and at the age-adjusted threshold (0.50 mg/L FEU up until age 50 years, and age × 0.01 mg/L FEU above age 50 years). The age-adjusted analysis was descriptive and was not applied as a prospective management algorithm. Threshold-specific sensitivity and specificity were reported with exact Clopper–Pearson 95% confidence intervals, and likelihood-ratio intervals were calculated on the logarithmic scale from 2 × 2 tables. Thresholding did not alter the AUROC of the underlying continuous D-dimer measurement.

Incremental value was evaluated with logistic models for D-dimer alone and D-dimer plus ET-1, as well as Wells alone, Wells plus D-dimer, Wells plus ET-1, and Wells plus D-dimer plus ET-1. D-dimer and ET-1 were log10-transformed and standardized to 1-SD units. Added discrimination was assessed by changes in AUROC using the paired DeLong test; nested-model fit was compared by likelihood-ratio testing. Paired stratified bootstrap resampling (2000 replicates) was used for confidence intervals around AUROC changes. For Wells plus D-dimer versus Wells plus D-dimer plus ET-1, continuous net reclassification improvement (NRI) and integrated discrimination improvement (IDI) were calculated with stratified bootstrap 95% confidence intervals. Because the selected study sample does not provide population-calibrated clinical risk categories, these reclassification metrics were considered exploratory and were not interpreted as measures of clinical utility. A separate exploratory model adjusted the ET-1 association for age, sex, malignancy, immobility, pre-existing cardiovascular disease, and log10-transformed CRP. Exploratory subgroup analyses summarized biomarker discrimination with DeLong AUROCs and used logistic regression with log10-transformed biomarker concentrations standardized to 1-SD units. Cardiovascular disease interaction models included biomarker concentration, cardiovascular disease status, and their product term; within-PE associations with binary RV dysfunction, PESI-based outcomes, and in-hospital mortality were estimated with univariable logistic regression, and outcome discrimination was summarized using AUROC. Correlations used Spearman’s rho. All subgroup and clinical-outcome analyses were hypothesis-generating.

Primary analyses used all available observations. Between-group biomarker comparisons were additionally summarized with Benjamini–Hochberg false-discovery-rate adjusted q-values across the four comparisons comprising D-dimer and the three candidate biomarkers; secondary cardiac markers were not included in that family. All tests were two-sided, and *p* < 0.05 was considered statistically significant. Given the exploratory design and multiple secondary analyses, emphasis was placed on effect estimates and confidence intervals rather than isolated *p*-values.

### 2.7. Sample Size

Because no clinically validated thresholds or robust prior AUROC estimates were available, sample size was based on a medium standardized between-group difference. A sample size calculation using G*Power (version 3.1.9.7; Heinrich Heine University Düsseldorf, Düsseldorf, Germany), with a two-sided α of 0.05, 80% power, and Cohen’s d of 0.5, indicated that 64 participants per group were required. The final sample included 65 PE-confirmed participants and 64 symptomatic imaging-negative controls. This study was not powered specifically for small AUROC differences or multivariable incremental-value analyses.

## 3. Results

Of the 140 enrolled participants, 11 were excluded because of missing data or a hemolyzed sample (5 in the PE group and 6 controls), leaving 129 participants: 65 with CTPA-confirmed PE and 64 symptomatic CTPA-negative controls (Figure 1). The median age was similar between groups (62 (47–74) vs. 61 (44–69) years; *p* = 0.344), as was the proportion of women (41.5% vs. 46.9%; *p* = 0.542). Malignancy and immobility were more frequent in the PE group, whereas pre-existing cardiovascular disease was more common in the controls. The presenting symptoms and clinical characteristics of both groups are shown in Table 1.

Chest pain, deep-vein thrombosis, and RV dysfunction on index transthoracic echocardiography were more frequent in the PE group. Echocardiography was available for all 129 participants; RV dysfunction was recorded in 30 of the 65 PE-confirmed participants and 3 of the 64 controls. PE-confirmed participants also had higher heart and respiratory rates, greater gas-exchange abnormalities, and higher CRP, AST, ALT, LDH, hs-troponin, and NT-proBNP concentrations. The Wells score was higher in the PE group (Table 1). BMI and obesity status, final diagnoses among controls, complete ESC risk categories, obstructive-shock counts, and use of systemic thrombolysis or other advanced reperfusion therapies were not available.

**Figure 2:**
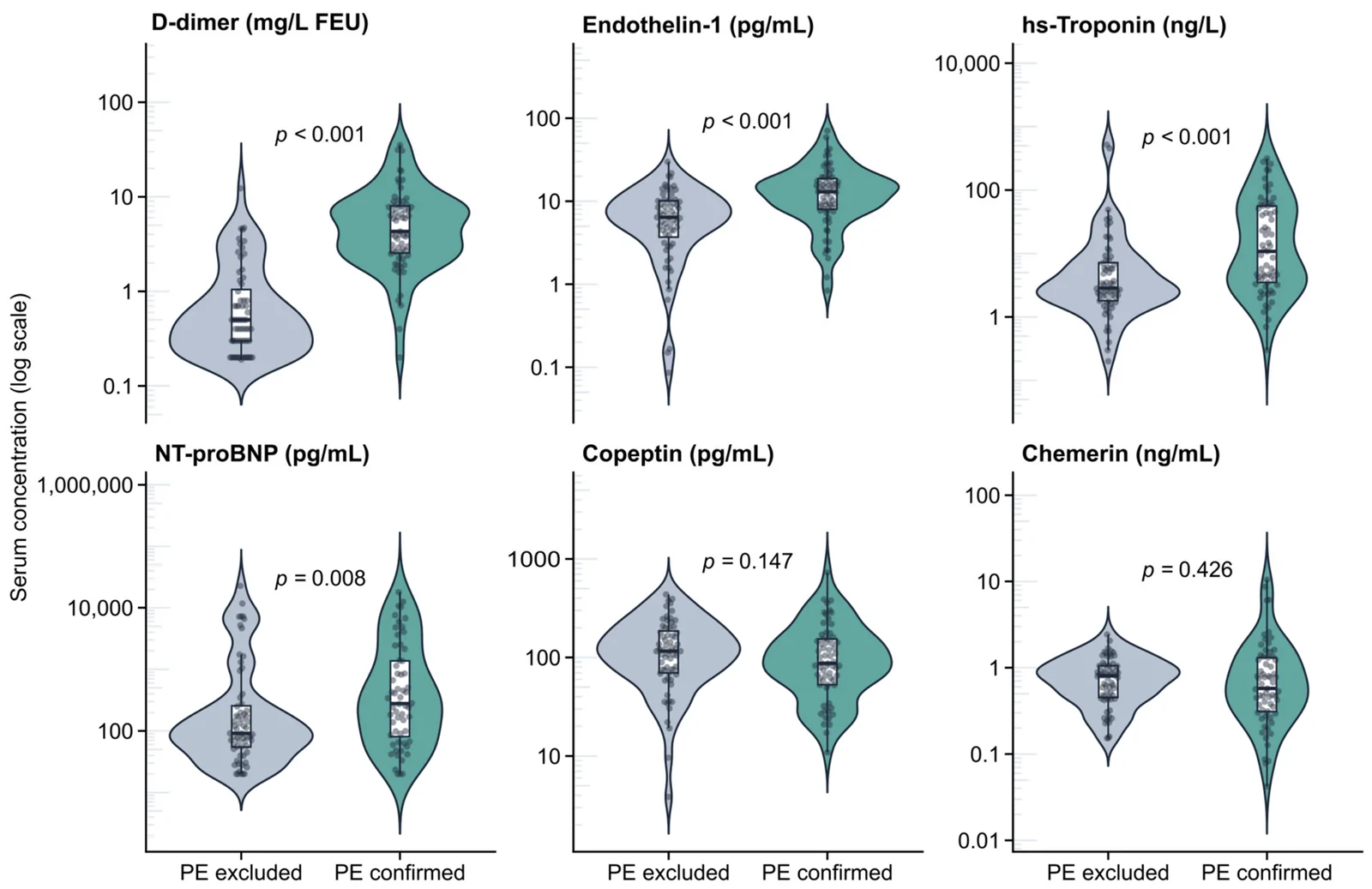
Biomarker concentrations according to pulmonary embolism status (*n* = 129). Note. Violin plots show biomarker distributions; boxes indicate medians and interquartile ranges, and points represent individual participants. Concentrations are displayed on a logarithmic scale. Candidate biomarkers were measured in serum; routine D-dimer was measured in plasma. The D-dimer axis is expressed in mg/L FEU. *p*-values are from Mann–Whitney U tests. PE excluded, *n* = 64; PE confirmed, *n* = 65.

The biomarker concentrations according to PE status are summarized in Table 2 and Figure 2. Among the three index biomarkers, endothelin-1 was higher in PE-positive patients (12.96 (8.01–18.75) vs. 6.41 (3.71–10.15) pg/mL), with a moderate positive effect size (r = 0.46; 95% CI, 0.27–0.63; *p* < 0.001; q < 0.001). Copeptin and chemerin did not differ significantly after false-discovery-rate correction: copeptin tended to be lower in patients (87.1 (53.0–153.7) vs. 115.8 (69.6–185.4) pg/mL; r = −0.15; 95% CI, −0.35–0.04; *p* = 0.147; q = 0.196), and chemerin was comparable between groups (0.57 (0.31–1.31) vs. 0.80 (0.45–1.05) ng/mL; r = −0.08; 95% CI, −0.30–0.12; *p* = 0.426; q = 0.426). D-dimer showed the strongest separation (4.30 (2.55–8.00) vs. 0.50 (0.30–1.05) mg/L FEU; r = 0.81; 95% CI, 0.70–0.91; *p* < 0.001; q < 0.001). Among the secondary cardiac markers, hs-troponin (10.8 (3.5–56.0) vs. 2.8 (1.8–7.2) ng/L; r = 0.41; *p* < 0.001) and NT-proBNP (278 (81–1369) vs. 91 (55–256) pg/mL; r = 0.28; *p* = 0.008) were higher in the patients (Table 2).

Eight ET-1 values (6.2%), 16 copeptin values (12.4%), and 10 chemerin values (7.8%) were outside the corresponding manufacturer-reported measurement ranges. Four values for each candidate biomarker were below the reported assay sensitivity; one chemerin value exceeded the upper measurement boundary. The conclusions were unchanged after boundary censoring (AUROCs: ET-1 0.730, copeptin 0.423, chemerin 0.461) and after excluding out-of-range observations (0.726, 0.459, and 0.472, respectively; Supplementary Table S5).

**Figure 3:**
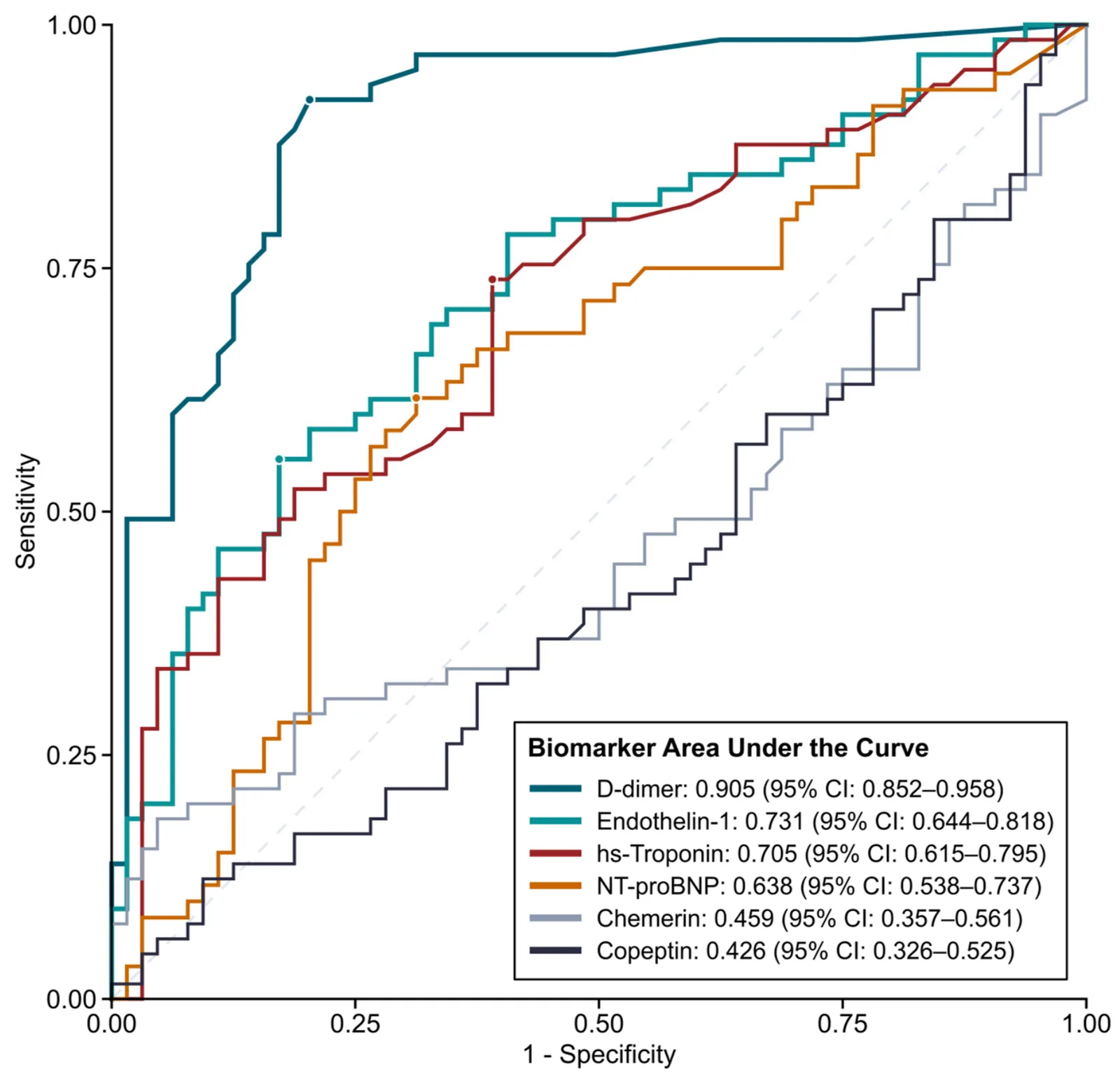
Receiver operating characteristic curves for discriminating confirmed from excluded pulmonary embolism. Note. Curves show D-dimer, ET-1, hs-troponin, NT-proBNP, chemerin, and copeptin for distinguishing CTPA-confirmed participants from symptomatic CTPA-negative controls. Areas under the curve use DeLong 95% confidence intervals. The dashed diagonal denotes no discrimination. The study sample comprised 129 participants: 65 with CTPA-confirmed PE and 64 symptomatic CTPA-negative controls.

D-dimer showed the highest continuous-marker diagnostic performance (AUROC, 0.905; 95% CI, 0.852–0.958). At the routine fixed threshold of 0.50 mg/L FEU, sensitivity was 96.9% (95% CI, 89.3–99.6) and specificity was 48.4% (95% CI, 35.8–61.3). The age-adjusted threshold preserved sensitivity at 96.9% (95% CI, 89.3–99.6) and increased specificity to 56.3% (95% CI, 43.3–68.6). At the exploratory Youden cutoff of 1.60 mg/L FEU, sensitivity was 92.3% (95% CI, 83.0–97.5) and specificity was 79.7% (95% CI, 67.8–88.7) (Supplementary Table S7). ET-1 showed moderate discrimination (AUROC, 0.731; 95% CI, 0.644–0.818) but was inferior to D-dimer (*p* < 0.001). At its exploratory 12.3 pg/mL threshold, sensitivity was 55.4% (95% CI, 42.5–67.7) and specificity was 82.8% (95% CI, 71.3–91.1). At hypothetical pre-test probabilities of 15% and 25%, a positive ET-1 result at this threshold would yield post-test probabilities of 36.2% and 51.8%, respectively; a negative result would yield 8.7% and 15.3%. These illustrative values are not observed predictive values in the selected study sample. Copeptin and chemerin were non-discriminative (AUROCs, 0.426 and 0.459), so cutoff-based operating characteristics were not emphasized (Table 3; Figure 3).

Adding ET-1 to D-dimer alone increased the AUROC from 0.905 (95% CI, 0.852–0.958) to 0.923 (95% CI, 0.875–0.970), a change of 0.0178 (paired DeLong *p* = 0.128; bootstrap 95% CI for the change, −0.0042 to 0.0431). The ET-1 coefficient in the augmented model was associated with PE status (OR per 1-SD increase in log10 ET-1, 2.31; 95% CI, 1.18–4.52; Wald *p* = 0.014), and nested-model fit improved (likelihood-ratio *p* = 0.008), but the improvement in rank discrimination was not statistically significant (Supplementary Table S3A).

The Wells score alone yielded a C-statistic of 0.941, and adding D-dimer increased it to 0.966. Against this high-discrimination baseline, adding ET-1 increased the C-statistic only to 0.971 (Δ = 0.0048; DeLong *p* = 0.224; bootstrap 95% CI for Δ, −0.0025 to 0.0137). Although the nested-model likelihood-ratio test yielded *p* = 0.041, the adjusted ET-1 coefficient was imprecise (OR per 1-SD increase in log10 ET-1, 2.29; 95% CI, 0.92–5.68; Wald *p* = 0.075). Continuous NRI was 0.572 (bootstrap 95% CI, −0.231 to 1.659) and IDI was 0.0232 (bootstrap 95% CI, 0.0001–0.0681). The wide NRI interval, absence of prespecified clinical risk categories, selected sampling frame, and near-ceiling comparator preclude interpreting these exploratory reclassification measures as clinical benefit (Supplementary Table S3A,B).

In an exploratory model adjusted for age, sex, malignancy, immobility, pre-existing cardiovascular disease, and log10 CRP, ET-1 remained associated with PE status (OR per 1-SD increase in log10 ET-1, 2.18; 95% CI, 1.09–4.33; *p* = 0.027; Supplementary Table S6). Subgroup analyses showed no ET-1-by-cardiovascular-disease interaction (*p* = 0.392; Supplementary Table S1). Within the PE-confirmed participants, hs-troponin and NT-proBNP were associated with right-ventricular dysfunction, whereas ET-1 was not (Supplementary Table S2).

Within the PE-confirmed participants, hs-troponin and NT-proBNP had higher exploratory AUROCs for high PESI classes and in-hospital mortality than the candidate diagnostic biomarkers; no formal between-marker comparison was performed (Supplementary Table S4). The mortality analyses included only eight events and are hypothesis-generating.

Spearman correlation analyses showed a strong correlation between hs-troponin and NT-proBNP (rho = 0.68) and a moderate correlation between D-dimer and CRP (rho = 0.59; Supplementary Figure S1). Among the PE-confirmed participants, hs-troponin and NT-proBNP correlated with right-ventricular dysfunction and PESI-based severity indicators, whereas ET-1 did not correlate significantly with the evaluated severity measures. These descriptive correlations do not establish distinct biological pathways.

## 4. Discussion

In this prospectively enrolled sample drawn from a common emergency-department source population, ET-1 was higher in participants with imaging-confirmed acute PE and showed moderate diagnostic discrimination. Copeptin and chemerin were non-discriminative, and D-dimer remained substantially stronger than ET-1. ET-1 did not materially improve AUROC when added to a Wells–D-dimer model. Because that baseline model already had a C-statistic of 0.966, little discriminative headroom remained for an additional marker. The incremental analysis should therefore be interpreted as a failure to demonstrate added clinical value in this selected design, not as proof that ET-1 adds no information in an unselected suspected-PE population.

The ET-1 signal is biologically coherent. Acute embolic obstruction increases pulmonary vascular resistance and right-ventricular afterload, creating endothelial shear stress, hypoxia, and disturbed pulmonary blood flow; a reduced perfused pulmonary vascular surface may also impair ET-1 clearance [9,10]. ET-1 overexpression occurs in pulmonary vascular disease, and experimental thromboembolism models show local ET-1 upregulation with hemodynamic improvement after receptor blockade [11,13]. Our findings extend this mechanistic rationale but should not be interpreted as establishing ET-1 as a diagnostic adjunct.

Our findings align with Sofia et al. [14], who reported increased arterial ET-1, an elevated transpulmonary ET-1 gradient, and higher urinary ET-1 excretion during acute PE, but contrast with Kostrubiec et al. [17], who found admission plasma ET-1 to be neither elevated nor prognostic. Related evidence in chronic thromboembolic pulmonary hypertension shows increased circulating and pulmonary microvascular ET-1-pathway activity [15] and reductions in circulating ET-1 after pulmonary endarterectomy [16]. These chronic-disease observations support biological plausibility but do not establish acute diagnostic performance. Differences in the sampling compartment, assay platform, timing, and study population may explain the discordant acute-PE findings. ET-1 assays are not directly interchangeable; our serum ELISA results should therefore be interpreted as an internally consistent discriminatory signal rather than as absolute values comparable with plasma-based studies.

The short circulating half-life, rapid receptor uptake, and limited stability of ET-1 complicate its interpretation as a single-time-point biomarker [31]. Human infusion studies demonstrate rapid initial plasma disappearance with substantial regional extraction followed by slower phases, rather than one universal half-life [32]. Immunoreactive ET-1 measurements are also sensitive to matrix, extraction, sample preparation, and assay specificity [33]. Circadian/time-of-day variation has been reported under controlled conditions [34], while small human studies indicate transient, stimulus-dependent changes with postural transition and different forms of exercise [35,36]. In this pragmatic emergency-department study, time of day, posture, preceding activity, and rest duration were not protocolized; these factors may have increased measurement variability but also reflect real-world emergency sampling. Big ET-1(1-38), which has been reported to have longer circulating phases and may better reflect endothelin-system secretory activity, warrants evaluation as a future pathway marker, without assuming superiority in acute PE [37].

ET-1 was not associated with right-ventricular dysfunction or PESI-based severity, whereas hs-troponin and NT-proBNP more clearly tracked myocardial stress. This pattern is compatible with, but does not prove, an endothelial response linked to PE presence. The covariate-adjusted association supports further study, while the minimal and non-significant AUROC gain beyond Wells plus D-dimer argues against current clinical adoption.

Copeptin showed no overall diagnostic value, consistent with studies using symptomatic emergency-department controls [23,24]. The tendency toward lower copeptin in PE may partly reflect group composition because pre-existing cardiovascular disease was more common among the controls (34.4% vs. 18.5%). Although a biomarker-by-cardiovascular-disease interaction was observed, copeptin remained non-discriminative in the cardiovascular-disease-free stratum and showed an inverse association in the smaller cardiovascular-disease stratum. These exploratory findings should not be taken as evidence of a clinically useful interaction and remain consistent with copeptin reflecting generalized neurohormonal stress rather than PE-specific biology [19,20,25,26].

Clinical evidence on chemerin as a diagnostic biomarker in acute PE remains very limited [4]. Although chemerin can promote endothelial NF-κB activation and monocyte–endothelial adhesion in vitro [29] and is linked to thrombo-inflammatory and metabolic pathways relevant to vascular disease [7,27,28], it did not differ between patients with and without PE in our cohort. Its vascular effects may be more relevant to chronic metabolic or atherosclerotic disease than to the acute embolic event, or any acute change may be too small or delayed to support emergency diagnosis. Within the analytical limitations described above, these data do not support chemerin as a stand-alone diagnostic marker for acute PE.

D-dimer remained the dominant biomarker comparator, and none of the candidate markers approached its continuous discrimination. The age-adjusted threshold preserved sensitivity and reduced false-positive results relative to the fixed 0.50 mg/L FEU threshold in this sample, consistent with its intended role in non-high-probability diagnostic pathways [1,38]. However, the threshold analysis was retrospective, was applied to the selected study sample, and was not a prospective rule-out strategy; it therefore cannot establish safety or imaging reduction. The difference between healthy-control and symptomatic-control studies underscores the importance of evaluating biomarkers in clinically relevant comparison groups [4]. At 55.4% sensitivity, ET-1 cannot support a rule-out role. Its value beyond established clinical assessment and D-dimer was not demonstrated here.

The unusually high discrimination of the Wells score in this sample (C-statistic = 0.941) should be interpreted in the context of the selected sample drawn from a common emergency-department source population. Spectrum restriction and the contrast between imaging-confirmed cases and consecutive dyspneic CTPA-negative controls likely produced a near-ceiling baseline model. With the Wells–D-dimer model already yielding a C-statistic of 0.966, little statistical headroom remained for detecting further improvement. Accordingly, the absence of a detectable increment with ET-1 should be regarded as inconclusive within this design rather than as definitive evidence of no incremental value.

### Strengths and Limitations

This study’s strengths include prospective specimen collection, imaging-based group classification, blinded biomarker analysis, and use of symptomatic imaging-negative participants rather than healthy volunteers. This study also compared candidate biomarkers directly with D-dimer and evaluated the clinically relevant Wells–D-dimer pathway.

Several limitations constrain interpretation. First, the single-center, approximately 1:1 analytical sample does not represent PE prevalence or the full clinical spectrum of an unselected emergency-department cohort. Both groups were drawn from the same emergency-department source population; controls were consecutive dyspneic patients evaluated for suspected PE in whom CTPA excluded PE. However, the precise stopping rule underlying the initially balanced groups was not available for verification, the study exclusions restricted the spectrum of alternative cardiopulmonary conditions, and final discharge diagnoses among the controls were unavailable. Second, the Wells score was recorded prospectively before CTPA interpretation, but its unusually high C-statistic (0.941) likely reflects the selected between-group contrast. The Wells–D-dimer model (0.966) created a near-ceiling comparator and limited power to detect small increments from ET-1; all Wells-based incremental and reclassification analyses require external validation. Third, this study was not powered for small AUROC increments or multivariable models, and thresholds were derived or evaluated in the same sample. Fourth, a small proportion of candidate-biomarker values lay outside their manufacturer-reported measurement ranges; although sensitivity analyses were concordant, absolute concentrations and cutoffs require confirmation with analytically validated assays. Fifth, the additional study blood tube was obtained before treatment, but the exact timing of routine D-dimer relative to treatment was not separately verified. Sampling time, posture, preceding activity, and rest were not standardized; detailed storage conditions and duration, freeze–thaw history, and lot- or plate-level quality-control information were unavailable. Sixth, height and weight were not collected; BMI and obesity status were unavailable, and residual adiposity-related confounding could not be excluded in the chemerin analysis. Seventh, although all participants underwent TTE and RV dysfunction was defined operationally by an RV/LV ratio ≥ 1.0 or TAPSE < 16 mm, only the binary RV-dysfunction status was retained; component values and source images could not be re-examined, and acute PE-related versus pre-existing dysfunction could not be fully adjudicated. Eighth, complete ESC risk-classification variables, obstructive-shock status, and systemic thrombolysis or other advanced reperfusion-therapy data were unavailable. Finally, PESI-based and mortality analyses were secondary, and the mortality analyses included only eight events.

## 5. Conclusions

ET-1 was associated with imaging-confirmed acute PE and showed moderate discrimination in this selected sample drawn from a common emergency-department source population, whereas copeptin and chemerin were non-discriminative. ET-1 remained inferior to D-dimer and did not significantly improve either D-dimer alone or the Wells–D-dimer model. Its sensitivity is insufficient for a rule-out role. Because the selected sampling frame and near-ceiling comparator limited inference about incremental value, external validation in an unselected diagnostic cohort with prospectively recorded clinical probability is required before clinical utility can be inferred.

## Figures and Tables

**Table 1:** Baseline clinical characteristics, presenting features, arterial blood gas parameters, laboratory findings, and clinical probability scores according to study group (*N* = 129).

Characteristic	Overall (*N* = 129)	PE Confirmed (*n* = 65)	Symptomatic CTPA-Negative (*n* = 64)	*p*-Value
Age, years	62 (46–71)	62 (47–74)	61 (44–69)	0.344
Female sex	57 (44.2)	27 (41.5)	30 (46.9)	0.542
**Comorbidities**				
Hypertension	45 (34.9)	19 (29.2)	26 (40.6)	0.175
Diabetes mellitus	25 (19.4)	11 (16.9)	14 (21.9)	0.477
Smoking	33 (25.6)	17 (26.2)	16 (25.0)	0.881
Malignancy	23 (17.8)	20 (30.8)	3 (4.7)	<0.001
Cardiovascular disease	34 (26.4)	12 (18.5)	22 (34.4)	0.040
Immobility	27 (20.9)	25 (38.5)	2 (3.1)	<0.001
Prior stroke	7 (5.4)	6 (9.2)	1 (1.6)	0.115
Prior PE	4 (3.1)	4 (6.2)	0 (0.0)	0.119
Atrial fibrillation	5 (3.9)	3 (4.6)	2 (3.1)	1.000
Coagulation disorder	1 (0.8)	0 (0.0)	1 (1.6)	0.496
**Presentation & findings**				
Dyspnea	126 (97.7)	62 (95.4)	64 (100.0)	0.244
Chest pain	58 (45.0)	37 (56.9)	21 (32.8)	0.006
Hemoptysis	9 (7.0)	7 (10.8)	2 (3.1)	0.164
DVT	21 (16.3)	19 (29.2)	2 (3.1)	<0.001
RV dysfunction on index TTE	33 (25.6)	30 (46.2)	3 (4.7)	<0.001
**Vitals & blood gas**				
Heart rate, bpm	87 (79–105)	95 (80–110)	82 (76–90)	<0.001
Respiratory rate, /min	17 (15–20)	18 (16–20)	16 (13–18)	<0.001
pH	7.43 (7.39–7.46)	7.44 (7.41–7.47)	7.42 (7.38–7.44)	0.003
pCO_2_, mmHg	34.8 (30.7–40.5)	31.9 (28.8–38.1)	37.5 (33.8–42.5)	<0.001
pO_2_, mmHg	76.6 (57.1–89.0)	68.0 (54.0–85.4)	83.2 (64.9–92.2)	0.009
HCO_3_^−^, mmol/L	23.4 (22.2–24.8)	23.2 (21.8–25.5)	23.8 (22.5–24.8)	0.350
SaO_2_ (ABG), %	94.0 (88.0–96.0)	91.4 (84.4–95.2)	94.8 (93.0–96.6)	<0.001
Lactate, mmol/L	1.7 (1.3–2.2)	1.7 (1.3–2.2)	1.9 (1.3–2.2)	1.000
**Laboratory**				
CRP, mg/dL	1.68 (0.37–5.99)	4.40 (2.44–9.25)	0.56 (0.31–1.36)	<0.001
Glucose, mg/dL	120 (100–143)	125 (104–143)	112 (96–141)	0.093
BUN, mg/dL	14.9 (10.8–21.5)	15.9 (11.2–22.9)	12.8 (10.8–19.3)	0.079
Creatinine, mg/dL	0.9 (0.8–1.1)	0.9 (0.8–1.1)	0.9 (0.8–1.1)	0.375
AST, U/L	23 (17–35)	30 (19–38)	20 (16–28)	0.010
ALT, U/L	22 (14–34)	25 (16–44)	18 (13–26)	0.014
LDH, U/L	282 (223–377)	322 (237–390)	250 (196–347)	0.008
CPK, U/L	72 (48–100)	60 (42–96)	80 (52–122)	0.051
CK-MB, U/L	19 (13–38)	18 (12–29)	20 (14–43)	0.067
hs-Troponin, ng/L	4.7 (2.3–23.1)	10.8 (3.5–56.0)	2.8 (1.8–7.2)	<0.001
Hemoglobin, g/dL	13.4 (11.3–14.5)	13.1 (10.6–14.1)	13.9 (12.2–14.9)	0.019
Hematocrit, %	39.2 (35.0–42.3)	38.1 (32.7–41.7)	39.8 (37.0–42.7)	0.106
Platelets, ×10^3^/µL	242 (188–298)	245 (177–298)	239 (197–295)	0.752
NT-proBNP, pg/mL	160 (61–850)	278 (81–1369)	91 (55–256)	0.008
**Clinical probability and severity scores**				
Wells score	1.0 (0.0–3.0)	3.0 (1.5–4.5)	0.0 (0.0–0.0)	<0.001
Modified Geneva score (PE group only)		7.0 (5.0–11.0)		
PESI score		89 (61–112)		
sPESI score		1.0 (0.0–2.0)		
**PESI risk class, n (%)**				
I		16 (24.6)		
II		14 (21.5)		
III		14 (21.5)		
IV		11 (16.9)		
V		10 (15.4)		

Note. Values are medians (interquartile range) or n (%). Continuous variables were compared using the Mann–Whitney U test; categorical variables used Pearson’s χ2 test without continuity correction or Fisher’s exact test, as appropriate. Modified Geneva, PESI, sPESI, and PESI risk classes were available only for PE-confirmed participants and were not used as between-group diagnostic comparators. ABG, arterial blood gas; ALT, alanine aminotransferase; AST, aspartate aminotransferase; BUN, blood urea nitrogen; CRP, C-reactive protein; DVT, deep-vein thrombosis; LDH, lactate dehydrogenase; PE, pulmonary embolism; PESI, Pulmonary Embolism Severity Index; RV, right ventricular; sPESI, simplified PESI.

**Table 2:** Biomarker concentrations and effect sizes according to pulmonary embolism status (available-case analysis).

Biomarker	PE Confirmed (*n* = 65)	CTPA-Negative Controls (*n* = 64)	r (95% CI)	*p*	q
**Primary biomarkers**					
Endothelin-1, pg/mL	12.96 (8.01–18.75)	6.41 (3.71–10.15)	+0.46 (0.27–0.63)	<0.001	<0.001
Copeptin, pg/mL	87.1 (53.0–153.7)	115.8 (69.6–185.4)	−0.15 (−0.35–0.04)	0.147	0.196
Chemerin, ng/mL	0.57 (0.31–1.31)	0.80 (0.45–1.05)	−0.08 (−0.30–0.12)	0.426	0.426
D-dimer, mg/L FEU	4.30 (2.55–8.00)	0.50 (0.30–1.05)	+0.81 (0.70–0.91)	<0.001	<0.001
**Secondary cardiac markers**					
hs-Troponin, ng/L	10.8 (3.5–56.0)	2.8 (1.8–7.2)	+0.41 (0.22–0.59)	<0.001	
NT-proBNP, pg/mL	278 (81–1369)	91 (55–256)	+0.28 (0.07–0.47)	0.008	

Note. Values are medians (interquartile range). Between-group comparisons used the Mann–Whitney U test. Effect sizes are rank-biserial correlations with 95% confidence intervals. The q-value is the Benjamani–Hochberg adjusted *p*-value across D-dimer and the three candidate biomarker comparisons; secondary cardiac markers are reported without q-values. CI, confidence interval; PE, pulmonary embolism.

**Table 3:** Diagnostic performance of biomarkers for discriminating CTPA-confirmed pulmonary embolism from symptomatic CTPA-negative controls (available-case analysis).

Biomarker	AUROC (95% CI)	*p* vs. D-Dimer	Cutoff	Sensitivity, % (95% CI)	Specificity, % (95% CI)	LR+ (95% CI)	LR− (95% CI)
D-dimer, mg/L FEU	0.905 (0.852–0.958)	Reference	1.60	92.3 (83.0–97.5)	79.7 (67.8–88.7)	4.54 (2.78–7.42)	0.10 (0.04–0.23)
Endothelin-1, pg/mL	0.731 (0.644–0.818)	<0.001	12.3	55.4 (42.5–67.7)	82.8 (71.3–91.1)	3.22 (1.80–5.76)	0.54 (0.40–0.72)
Copeptin, pg/mL	0.426 (0.326–0.525)	<0.001					
Chemerin, ng/mL	0.459 (0.357–0.561)	<0.001					
hs-Troponin, ng/L	0.705 (0.615–0.795)	<0.001	3.9	73.8 (61.5–84.0)	60.9 (47.9–72.9)	1.89 (1.35–2.65)	0.43 (0.27–0.68)
NT-proBNP, pg/mL	0.638 (0.538–0.737)	<0.001	184	61.7 (48.2–73.9)	68.8 (55.9–79.8)	1.97 (1.30–2.99)	0.56 (0.39–0.80)

Note. Values are point estimates (95% CIs). AUROC confidence intervals were estimated using the DeLong method. *p*-values compare each biomarker AUROC with D-dimer using the correlated DeLong test. Confidence intervals for sensitivity and specificity were calculated using the exact Clopper–Pearson method, and those for likelihood ratios using the log method. Cutoffs were derived by maximizing Youden’s J statistic in the same sample and are exploratory. AUROC, area under the receiver operating characteristic curve; CI, confidence interval; LR+, positive likelihood ratio; LR−, negative likelihood ratio.

## Data Availability

The data that support the findings of this study are available from the corresponding author upon reasonable request.

## Supplementary Materials

The following supporting information can be downloaded at https://www.mdpi.com/article/10.3390/jcdd13090448/s1, Table S1: Exploratory biomarker performance according to cardiovascular disease status; Table S2: Exploratory performance by right-ventricular dysfunction; Table S3A: Incremental value of ET-1 in D-dimer- and Wells-based models; Table S3B: Exploratory continuous NRI and IDI; Table S4: Exploratory PESI- and mortality-based analyses; Table S5: Assay-range sensitivity analyses; Table S6: Exploratory multivariable analysis; Table S7: D-dimer operating characteristics at fixed, age-adjusted, and Youden thresholds; and Figure S1: Spearman correlation matrices for biomarkers and clinical severity measures.
