# Supplementary material for: Serum Endothelin-1 in Suspected Acute Pulmonary Embolism: A Prospective Study
Source: J Cardiovasc Dev Dis. 2026 Sep 9;13(9):448. doi: 10.3390/jcdd13090448 (PMC13607929; doi:10.3390/jcdd13090448)
Supplement: Supplementary file 1 [file jcdd-13-00448-s001.zip › jcdd-4506788-supplementary.pdf]

Supplementary Material

Serum Endothelin-1 in Suspected Acute Pulmonary Embolism: A Prospective Study

Supplementary Table S1. Exploratory biomarker diagnostic performance according to pre-existing cardiovascular disease status

Biomarker	Full cohort (n = 129)	CVD- stratum (n = 95)		CVD+ stratum (n = 34)		P for biomarker × CVD interaction
	AUROC (95% CI)	AUROC (95% CI)	OR per 1-SD (95% CI)	AUROC (95% CI)	OR per 1-SD (95% CI)	
D-dimer, mg/L FEU	0.905 (0.852-0.958)	0.923 (0.863-0.984)	13.33 (5.37-33.1)	0.913 (0.820-1.000)	20.35 (2.36-175.8)	0.422
Endothelin-1	0.731 (0.644-0.818)	0.766 (0.671-0.861)	3.10 (1.64-5.9)	0.652 (0.454-0.849)	2.07 (0.83-5.2)	0.392
hs-Troponin	0.705 (0.615-0.795)	0.796 (0.707-0.885)	4.73 (2.29-9.8)	0.530 (0.315-0.745)	1.16 (0.58-2.3)	0.008
NT-proBNP	0.638 (0.538-0.737)	0.759 (0.660-0.859)	4.06 (2.01-8.2)	0.487 (0.275-0.699)	0.90 (0.45-1.8)	0.001
Copeptin	0.426 (0.326-0.525)	0.481 (0.363-0.599)	1.05 (0.70-1.6)	0.254 (0.081-0.426)	0.34 (0.13-0.9)	0.030
Chemerin	0.459 (0.357-0.561)	0.445 (0.328-0.562)	0.85 (0.57-1.3)	0.532 (0.300-0.765)	1.25 (0.62-2.6)	0.356

Note. Values are AUROC with DeLong 95% confidence intervals and odds ratios per 1-SD increase in log10-transformed concentration. The CVD-negative stratum comprised 53 PE-positive and 42 PE-negative participants; the CVD-positive stratum comprised 12 and 22, respectively. For NT-proBNP, the CVD-negative stratum included 90 available participants (48 PE-positive/42 PE-negative) and the CVD-positive stratum included 34 (12/22). Interaction p values came from models containing biomarker, CVD, and biomarker × CVD terms and are exploratory. All biomarkers used n = 129. AUROC, area under the receiver operating characteristic curve; CI, confidence interval; CVD, cardiovascular disease; OR, odds ratio; PE, pulmonary embolism.

Supplementary Table S2. Exploratory biomarker performance by right ventricular dysfunction status among patients with pulmonary embolism

Biomarker	PE with RV dysfunction vs controls (30 + 64; total n = 94)		PE without RV dysfunction vs controls (35 + 64; total n = 99)		P for RV association
	AUROC (95% CI)	OR per 1-SD (95% CI)	AUROC (95% CI)	OR per 1-SD (95% CI)	
D-dimer, mg/L FEU	0.943 (0.900-0.985)	22.48 (6.00-84.2)	0.873 (0.799-0.947)	6.14 (3.09-12.2)	0.029
Endothelin-1	0.730 (0.608-0.851)	2.55 (1.31-5.0)	0.733 (0.630-0.835)	3.63 (1.75-7.5)	0.540
hs-Troponin	0.792 (0.683-0.901)	3.36 (1.92-5.9)	0.630 (0.516-0.745)	1.49 (0.97-2.3)	0.004
NT-proBNP	0.744 (0.631-0.857)	2.36 (1.46-3.8)	0.544 (0.421-0.667)	1.02 (0.67-1.6)	0.002
Copeptin	0.474 (0.336-0.613)	0.94 (0.61-1.4)	0.384 (0.272-0.497)	0.74 (0.49-1.1)	0.373
Chemerin	0.461 (0.322-0.601)	0.95 (0.61-1.5)	0.457 (0.327-0.587)	0.88 (0.59-1.3)	0.835

Note. Index transthoracic echocardiography was performed in all 129 analyzed participants. RV dysfunction was defined operationally as an end-diastolic right ventricular-to-left ventricular (RV/LV) diameter ratio ≥1.0 or tricuspid annular plane systolic excursion (TAPSE) <16 mm; either criterion was sufficient when documented. Free-wall hypokinesia, septal flattening, and the McConnell sign were supportive findings only and were not sufficient alone. The analytical dataset retained only the resulting binary RV-dysfunction status and not the individual numeric RV/LV or TAPSE values or source images for central re-reading. Therefore, the component thresholds could not be re-applied or independently checked for individual participants. Prior echocardiograms were not systematically available, so the binary endpoint could not always distinguish acute PE-related RV strain from pre-existing RV dysfunction. RV dysfunction was present in 30 of 65 PE patients and absent in 35; it was recorded in 3 of 64 controls. Each AUROC shown compares the stated PE subgroup with the same 64 symptomatic CTPA-negative controls, irrespective of control RV status. The final column tests the biomarker-RV dysfunction association within PE patients using separate univariable logistic models; odds ratios and p values are reported per 1-SD increase in log10-transformed biomarker concentration. All other biomarkers had complete data. Echocardiographers were unaware of subsequently measured research-biomarker results but had access to routine clinical information. D-dimer was recorded in mg/L FEU. These subgroup analyses are exploratory. AUROC, area under the receiver operating characteristic curve; CI, confidence interval; CTPA, computed tomographic pulmonary angiography; FEU, fibrinogen-equivalent units; OR, odds ratio; PE, pulmonary embolism; RV, right ventricular; RV/LV, right ventricular-to-left ventricular; TAPSE, tricuspid annular plane systolic excursion.

Supplementary Table S3A. Incremental diagnostic value of ET-1 in D-dimer- and Wells-based models

Model	n	ET-1 OR per 1-SD (95% CI)	Augmented AUROC (95% CI)	Comparator AUROC	ΔAUROC (bootstrap 95% CI)	DeLong p	Likelihood-ratio p
D-dimer	129	—	0.905 (0.852-0.958)	—	—	—	—
D-dimer + ET-1	129	2.31 (1.18-4.52)	0.923 (0.875-0.970)	0.905	+0.0178 (-0.0042 to 0.0431)	0.128	0.0080
Wells	129	—	0.941 (0.899-0.982)	—	—	—	—
Wells + D-dimer	129	—	0.966 (0.933-0.999)	0.941	+0.0257	0.0418	<0.001
Wells + ET-1	129	2.79 (1.28-6.10)	0.959 (0.925-0.994)	0.941	+0.0188	0.0467	0.0021
Wells + D-dimer + ET-1	129	2.29 (0.92-5.68)	0.971 (0.941-1.000)	0.966	+0.0048 (-0.0025 to 0.0137)	0.224	0.0407

Note. Logistic regression modeled CTPA-confirmed PE versus symptomatic CTPA-negative controls. D-dimer was recorded in mg/L FEU; D-dimer and ET-1 were log10-transformed and standardized; the ET-1 odds ratio is adjusted for the other terms in the row. Paired AUROCs were compared with the DeLong test, and likelihood-ratio p values compare nested models. Confidence intervals for the two reviewer-requested ΔAUROC comparisons were obtained from 2,000 paired stratified bootstrap samples using the fixed fitted-model scores. Although ET-1 improved nested-model fit when added to D-dimer alone, the AUROC increment was not statistically significant. Wells-based results remain exploratory and require external validation. AUROC, area under the receiver operating characteristic curve; CI, confidence interval; ET-1, endothelin-1; OR, odds ratio; PE, pulmonary embolism.

Supplementary Table S3B. Exploratory reclassification metrics for adding ET-1 to Wells plus D-dimer

Comparison	n	Continuous NRI	Bootstrap 95% CI	IDI	Bootstrap 95% CI
Wells + D-dimer vs. Wells + D-dimer + ET-1	129	0.572	-0.231 to 1.659	0.0232	0.0001 to 0.0681

Note. Continuous NRI and IDI compare the fitted probabilities from Wells plus D-dimer with those from Wells plus D-dimer plus ET-1. Confidence intervals used 2,000 stratified bootstrap samples with model refitting. No clinical risk categories were prespecified because the selected study sample does not yield population-calibrated risks. These metrics are post hoc, vulnerable to optimism, and should not be interpreted as evidence of clinical utility. CI, confidence interval; ET-1, endothelin-1; IDI, integrated discrimination improvement; NRI, net reclassification improvement.

Supplementary Table S4. Exploratory biomarker discrimination of PESI-based risk and in-hospital mortality among patients with pulmonary embolism

Biomarker	High PESI class III-V vs I-II		sPESI ≥1 vs 0		In-hospital mortality	
	AUROC (95% CI)	OR per 1-SD (95% CI)	AUROC (95% CI)	OR per 1-SD (95% CI)	AUROC (95% CI)	OR per 1-SD (95% CI)
D-dimer, mg/L FEU	0.692 (0.562-0.823)	2.47 (1.30-4.69)	0.556 (0.410-0.703)	1.32 (0.79-2.21)	0.700 (0.462-0.937)	2.73 (1.07-6.93)
Endothelin-1	0.526 (0.383-0.669)	0.97 (0.60-1.58)	0.654 (0.517-0.792)	1.53 (0.90-2.58)	0.553 (0.270-0.835)	1.07 (0.50-2.27)
hs-Troponin	0.751 (0.631-0.872)	2.90 (1.55-5.40)	0.650 (0.500-0.799)	1.81 (1.03-3.17)	0.860 (0.753-0.966)	5.97 (1.73-20.60)
NT-proBNP	0.764 (0.634-0.893)	2.75 (1.43-5.29)	0.667 (0.522-0.812)	1.93 (1.06-3.52)	0.781 (0.643-0.919)	2.60 (1.14-5.94)
Copeptin	0.402 (0.262-0.542)	0.74 (0.45-1.22)	0.468 (0.321-0.615)	0.86 (0.51-1.43)	0.325 (0.071-0.578)	0.49 (0.22-1.09)
Chemerin	0.528 (0.381-0.675)	1.04 (0.64-1.70)	0.590 (0.441-0.738)	1.35 (0.79-2.29)	0.592 (0.376-0.808)	1.47 (0.70-3.08)

Note. Analyses were restricted to imaging-confirmed PE (n = 65). Biomarker concentrations were log10 transformed and standardized; odds ratios are reported per 1-SD increase from separate univariable logistic models. High PESI class III-V was present in 35 patients, sPESI ≥1 in 42, and in-hospital death in 8. All analyses are exploratory, and mortality estimates are particularly uncertain because of the small event count. AUROC, area under the receiver operating characteristic curve; CI, confidence interval; OR, odds ratio; PE, pulmonary embolism; PESI, Pulmonary Embolism Severity Index; sPESI, simplified PESI.

Supplementary Table S5. Recorded assay-range status and sensitivity analyses

Biomarker	< LOD	LOD to < lower range	Within range	> upper range	Recorded-value AUROC	Floor/cap AUROC	In-range-only AUROC
Endothelin-1	4	4	121	0	0.731	0.730	0.726 (n = 121)
Copeptin	4	12	113	0	0.426	0.423	0.459 (n = 113)
Chemerin	4	5	119	1	0.459	0.461	0.472 (n = 119)

Note. Manufacturer-reported limits of detection and measurement ranges were 18.75 and 31.25-2,000 pg/mL for copeptin, 0.75 and 1.25-80 pg/mL for ET-1, and 0.10 and 0.16-10 ng/mL for chemerin. Recorded-value analyses used the numeric values in the analysis dataset. Floor/cap analyses censored values at the nearest measurement-range boundary. In-range-only analyses excluded values outside the stated range. These analyses assess robustness only and do not replace validation against raw optical-density values, standard curves, and laboratory quality-control records. AUROC, area under the receiver operating characteristic curve; LOD, limit of detection.

Supplementary Table S6. Exploratory multivariable model for imaging-confirmed pulmonary embolism

Variable	Adjusted OR (95% CI)	Wald p
Endothelin-1, per 1-SD log10 increase	2.18 (1.09-4.33)	0.027
Age, per 10-year increase	1.20 (0.88-1.64)	0.245
Female sex	0.40 (0.13-1.22)	0.106
Malignancy	7.59 (1.26-45.69)	0.027
Immobility	12.72 (2.16-75.03)	0.005
Pre-existing cardiovascular disease	0.17 (0.04-0.71)	0.014
CRP, per 1-SD log10 increase	3.68 (1.98-6.86)	<0.001

Note. The outcome was imaging-confirmed PE versus symptomatic imaging-negative control status (65 events among 129 participants). Continuous variables were standardized as indicated. This post hoc model contains seven predictors and is vulnerable to overfitting, collinearity, and spectrum/selection bias; the coefficients should therefore be interpreted as sensitivity analyses rather than confirmatory causal estimates. CI, confidence interval; CRP, C-reactive protein; OR, odds ratio; PE, pulmonary embolism.

Supplementary Table S7. D-dimer operating characteristics at fixed, age-adjusted, and exploratory Youden thresholds

Threshold	TP/FN/FP/TN	Sensitivity, % (95% CI)	Specificity, % (95% CI)	LR+ (95% CI)	LR- (95% CI)
Fixed ≥0.50 mg/L FEU	63/2/33/31	96.9 (89.3-99.6)	48.4 (35.8-61.3)	1.88 (1.48-2.39)	0.06 (0.02-0.25)
Age-adjusted threshold, mg/L FEU	63/2/28/36	96.9 (89.3-99.6)	56.3 (43.3-68.6)	2.22 (1.67-2.93)	0.05 (0.01-0.22)
Youden ≥1.60 mg/L FEU	60/5/13/51	92.3 (83.0-97.5)	79.7 (67.8-88.7)	4.54 (2.78-7.42)	0.10 (0.04-0.23)

Note. Analysis included all 129 participants (65 CTPA-confirmed PE and 64 symptomatic CTPA-negative controls). The age-adjusted threshold was 0.50 mg/L FEU through age 50 years and age × 0.01 mg/L FEU above age 50 years. It was applied retrospectively and descriptively, not as a prospective management rule. The continuous D-dimer AUROC was 0.905 (95% CI, 0.852-0.958); applying thresholds does not create a separate continuous-marker AUROC. The Youden threshold was selected and evaluated in the same sample and is optimistic. CI, confidence interval; FEU, fibrinogen-equivalent units; FN, false negative; FP, false positive; LR, likelihood ratio; TN, true negative; TP, true positive.

Supplementary Figure S1. Spearman correlation matrices for biomarkers and clinical severity measures.

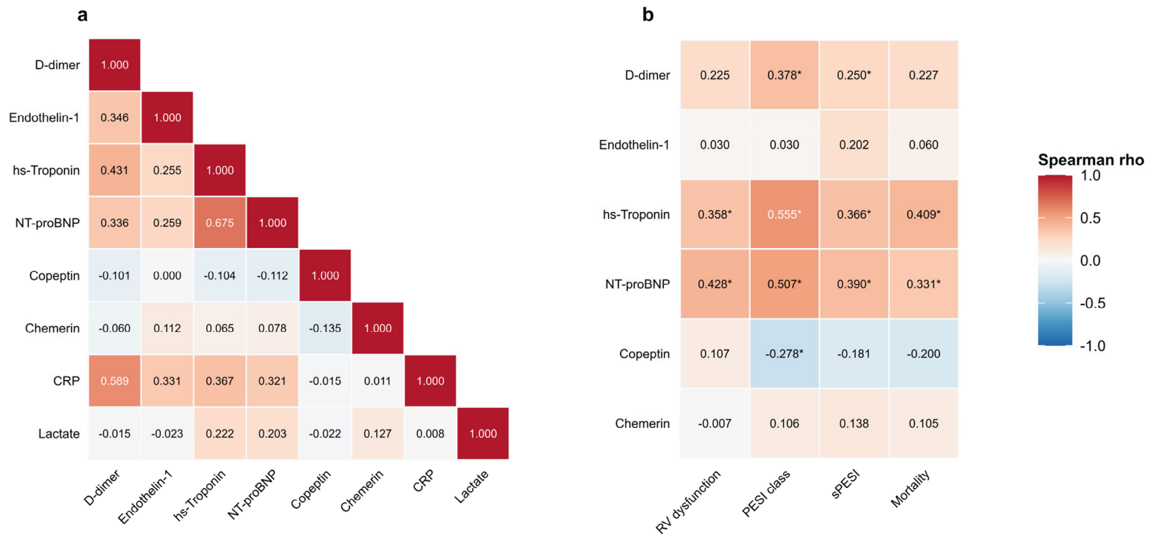

Note. Heatmaps display Spearman rank correlation coefficients (ρ) for (a) pairwise associations among biomarkers and (b) associations of biomarkers with right ventricular dysfunction, PESI class, sPESI, and mortality. Cell values indicate correlation coefficients; red and blue represent positive and negative correlations, respectively, with greater color intensity indicating stronger correlations. In panel (b), asterisks indicate $p < 0.05$. CRP, C-reactive protein; hs-Troponin, high-sensitivity troponin; NT-proBNP, N-terminal pro–B-type natriuretic peptide; PESI, Pulmonary Embolism Severity Index; RV, right ventricular; sPESI, simplified Pulmonary Embolism Severity Index.
